# Early infant feeding and allergic respiratory diseases in Ibb city, Yemen

**DOI:** 10.1186/s40001-022-00662-7

**Published:** 2022-03-03

**Authors:** Jamil M. A. S. Obaid, Waheed A. M. Ali, Antar F. A. M. Al-badani, Zakaria M. Damag, Tariq A. Aziz, Yosef M. Al-Ansi, Khawla A. Sadek

**Affiliations:** 1 Medical Laboratory Sciences Dept., Faculty of Medicine and Health Sciences, Ibb University, Ibb, Yemen; 2 Medical Microbiology Dept., Faculty of Science, Ibb University, Ibb, Yemen; 3 Medical Laboratory Sciences Dept., Faculty of Medicine and Health Sciences, Taiz University, Ibb, Yemen; 4 Pediatrics Department, Childhood and Maternity Hospital, Ibb, Yemen

**Keywords:** Allergy, Asthma, IgE, Risk factor, Infant feeding, Artificial milk

## Abstract

**Background:**

Allergic respiratory diseases (ARD) are a highly prevalent health problem affecting infants and children in Yemen. Early infant feeding predisposition to the development of ARD has been a controversial question. The aim of this study is to investigate the association between early feeding before 6 months of age and the development of ARD among children attending Childhood and Maternity Public Hospital (CMPH), Ibb, Yemen Republic.

**Subjects and methods:**

The study population included 151 child patients attending the pediatric clinic at CMPH. Upon clinical and laboratory examinations, 72 out of 151 patients had ARD, while the other 79 had diseases other than ARD; all of them were used in risk assessment. Fifteen blood samples from healthy volunteers were used in laboratory investigations as a control. Complete blood count and IgE level were investigated for all participants. Children's parents were requested to give an informed consent and fill questionnaire about demography and history details.

**Results:**

Early infant feeding was a significant risk factor for the development of ARD with an odds ratio (OR) of 6.8 and 95% confidence interval (CI) 3.0 to 15.3. Artificial milk particularly was risk factor with an OR of 6.1 and 95% confidence interval 2.7 to 13.5. Artificial milk exhibited more wheezing and asthma attack than others (OR 4.3, 95% CI 1.9 to 9.4 and OR 7.6, 95% CI 3.5 to 16.3, respectively). The risk of wheezing and asthma attack also increase with early feeding generally (OR 3.0, 95% CI 1.3 to 7.2 and OR 4.8, 95% CI 2.2 to 8.1, respectively). The patients had a higher sensitization markers than the control, such as eosinophil count and total serum IgE. The highest levels of IgE ever reported existed among early fed patients with artificial milk.

**Conclusions:**

Early infant feeding, particularly with artificial milk, is a risk factor predisposing infants to the development of allergic respiratory disease presented with more clinical features of wheezing and asthma attack.

## Background

Allergic respiratory diseases are major health problems among infants and children worldwide. Their prevalence increases with a time and it may reach as higher levels as 40% in some countries [1]. Yemen Republic is one of developing countries that lacks data of many diseases including ARD. One study about ARD in Yemen showed a prevalence of asthma and hay fever of 14.4%, 12%, respectively [2, 3]. The prevalence of childhood atopic diseases in general including eczema and food allergy, has increased in the recent decade [4]. Both the prevalence and the burden of allergic diseases are considerable exhibiting prevalence varying between 1 and 20% [5]. The impact of allergic respiratory diseases on the social, economic state and the individual quality of life requires special attention by health authorities [6]. Approximately 10% of children without an allergic parent or sibling, and 20–30% of those with allergies in their first-degree relatives, experience allergic diseases in infancy that may indicate to other non-hereditary environmental factors [7].

Allergic respiratory diseases are disorders of the airway characterized by hyper-responsiveness and inflammation manifested by symptoms ranging from as mild as sneezing to chronically severe such as asthma. Allergic rhinitis and asthma are the most common chronic childhood respiratory diseases worldwide [8]. The common histopathology findings include airway inflammatory cell infiltration such as eosinophils, neutrophils, and lymphocytes (especially T cells), goblet cell hyperplasia, sometimes plugging of small airways with mucus, airway edema, and mast cell activation. IgE plays a central role in the pathogenesis of allergic asthma [9].

The development of IgE-mediated allergic respiratory diseases is influenced by many factors; the first and most important one is genetic. Second factor may be the environmental factors such as pollution, climate, and nutrition. Nutritional factor effect was proven by the conclusion that the obesity is increasingly recognized as a risk factor. Continuous exposure of sensitized patients to inhaled allergens increases airway inflammation, airway hyper-responsiveness, and symptoms of atopy [10]. Continuous exposure to other environmental risk factors must be investigated.

Infant feeding is basically an important early-life exposure that may influence respiratory infections and the development of allergic respiratory diseases notably asthma [11, 12]. A large meta-analysis of early infant feeding and health outcomes showed lower impact of breast-feeding on cough and wheeze in a dose–response pattern [13]. By the way, World Health Organization (WHO) recommend early breast-feeding at first 1 h of birth and to be exclusive for 6 months, then continue for up to 2 years of age [14].

It is noteworthy to mention that the early-life nutrition is an important modifiable lifestyle factor that influences the development of a child’s immune system, consequently it may affect positively or negatively on immune response, i.e., it may lead to development of immune diseases [15]. Our hypothesis postulates that early feeding (before 6 months of age) may predispose to the development of allergic respiratory disease.

## Subjects and methods

### Subjects

This case–control study was conducted between January and May 2019 on 151 child patients. These patients were admitted to Pediatrics Clinic at Childhood and Maternity Public Hospital, Ibb, Yemen Republic (CMPH). Upon clinical and laboratory examinations, a total of 72 patients were diagnosed as having allergic respiratory disease with recurrent episodes of allergy according to the clinical guidelines. Seventy-nine children diagnosed with disease other than ARD were included for risk assessment analyses. Fifteen healthy children were included in this study as a control for laboratory investigations.

Patients’ parents were asked to get a full history and medical information to complete the questionnaire by our team with the help of patient’s physician. Double blood sample was taken from patient, one EDTA sample that was used for complete blood count analyses. Another clotted sample was used for serum IgE measurement using ELlSA technique. An informed consent for participation was taken for all participant on behalf of their parents. This research adheres to international guidelines of ethics in biomedical research.

### Questionnaire

The questionnaire was filled by researchers and parents and includes patient demography, and full medical history. Assessment of allergic respiratory disease data was taken from their response on questions about disease; first presentation, severity, number of episodes, medical assistance-called, history of hospital admissions, and symptoms. Activities and living habits related to hypersensitivity were included as well. The feeding items questions were included in questionnaire such as breast-feeding data, artificial milk consumption and other formula of juice and solid food in relation to patient age.

Eight non-compliant patients who gave incomplete information of questionnaire were excluded from this study.

### Complete blood count

EDTA blood sample was collected from each patient and healthy control volunteers. Samples were analyzed using hematology analyzer (Sysmex xsi-500, Germany) with 5 differential leukocytes parameters output. All CBC parameters were collected and statistically analyzed.

### IgE determination

Immunoglobulin E was measured from frozen serum samples of patients and controls. The assay is based on a solid-phase enzyme-linked immunosorbent assay (ELISA) system that utilizes one monoclonal anti-IgE antibody for solid-phase (micro-titer wells) immobilization. Further procedures followed the standard protocol of the manufacturer (Bios, Hayward CA, USA). Color absorbance was measured with ELISA reader (Mindary, China) in reference to standard reagents.

### Statistical analysis

All statistical analyses were conducted using IBM SPSS version 19 (property of SPSS 2010, Inc., IBM Company) software. The difference tests for categorical variables were carried out using Chi-square test, and *t* test for quantitative variables. Correlation analyses were carried out with Spearman correlation test. Risk factor assessment was done using odds ratio (OR) calculation with 95% confidence intervals (CI) from ARD patients and other patients distribution table using SigmaPlot for windows version 12, GmbH, Germany. The *p* value less than 0.05 was considered statistically significant.

## Results

A total of 151 child patients were selected to take part in this study. Out of the 151, 72 were diagnosed with ARD. Their age ranged between 2 months and 15 years; 61% of them were males. The majority of patients inhabit rural areas (68%). Those who live at the ground floor were 64% and those who had pets at home were 26%. All these data and other demographic data are listed in Table 1.

**Table 1:** Demographic data of ARD patients and control volunteers attending Children and Maternity Hospital, Ibb, Yemen

Data	Patients (percentage)	Control (percentage)
Sex		
Male	61	60
Female	39	40
Age groups		
0–6 m	14	7
7 m–2	32	33
2.1–7	42	40
7–15	13	20
Residence		
Urban	32	27
Rural	68	73
Income		
High	18	13
Intermediate	51	33
Low	32	53
Home inhabited-floor		
1	64	47
2	21	40
3 or more	15	13
Breast-feeding		
Yes	93	87
No	7	13
Pets at home		
Yes	26	27
No	74	73
No. of persons sharing the room		
1	8	20
2	31	27
3	32	40
4 or more	29	13

The red blood cell parameters were lower than that of the healthy control (Table 2). In contrast, the mean platelets count and total leukocytes count were higher than that of the control with a statistical significance of *p* < 0.001. Lymphocytes and eosinophils were elevated as well. The mean eosinophil count of ARD patient was four times greater than that of healthy counterparts (*p* < 0.001).

**Table 2:** Complete blood cell count means of ARD patients and healthy control

	Hb	PCV	MCV	MCH	MCHC	PLTs	WBC	N	L	E
Patients										
Mean	11.2	35.9	79.1	24.8	30.6	396.8	8.9	3.5	4.3	0.45
Range	7.4–15.0	24.4–43.5	55.2–98.8	14.4–32.4	21.2–35.6	189.0–788.0	3.5–17.7	1.4–12.4	1.2–10.3	0.04–1.32
Control										
Mean	12.7	37.3	76.4	26.8	34.1	270.0	6.4	4.4	1.8	0.10
Range	7.3–16.6	21.4–66.6	58.7–86.7	20.8–31.4	30.0–36.3	84.0–400.0	2.8–12.3	1.8–10.0	0.3–4.5	0.03–0.26
*p* value	0.009	0.782	0.173	0.014	< 0.001	< 0.001	< 0.001	0.151	< 0.001	< 0.001

*Hb* hemoglobin, *PCV* packed cell volume, *MCV* mean cell volume, *MCH* mean cell hemoglobin, *MCHC* mean cell hemoglobin concentration, *PLTs *platelets, *WBCs *white blood cells, *N* neutrophils, *L* lymphocytes, *E* eosinophils

**Fig. 1:**
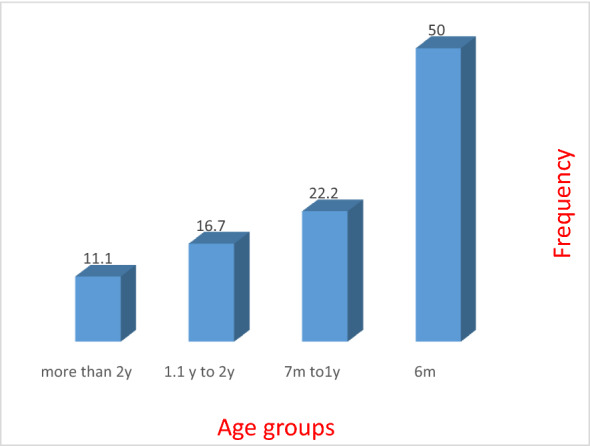
Age of disease presentation for ARD patients

**Fig. 2:**
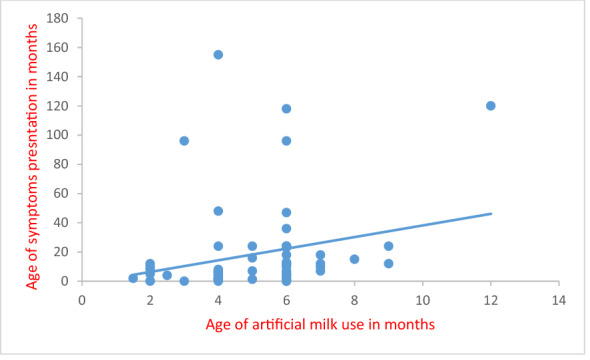
Correlation between first age of artificial milk use and age of ARD symptoms presentation

More than half of patients developed ARD below 6 months of age. By the age of 2 years, 85% of patients developed ARD (Fig. 1). Patients presented with severe symptoms to the degree that need medical assistance scored a percentage of 46%, and about 68% were admitted to the hospital and were put under therapeutic sessions with oxygen and nebulizer (Table 3). The association between early feeding in general and artificial milk with hospital admission showed odds ratio (OR 3.1, 95% CI 1.5 to 6.7 and OR 2.2, 95% CI 1.1 to 4.7, respectively). The association between early feeding in general and artificial milk with medication showed odds ratio (OR 4.9, 95% CI 2.2 to 10.8 and OR 4.8, 95% CI 2.2 to 10.4, respectively). The age of disease presentation is directly proportional to the age at which the child started artificial milk feeding (*r* = 0.482, *p* = 0.005) as depicted in Fig. 2.

**Table 3:** Medical assistance need and medications history

Data	Response	Percentage
Medical assistance need	Yes	45.8
	No	54.2
Admission to hospital	Yes	69.4
	No	30.6
Medication	Yes	68.1
	No	31.9
Nebulizer use	Yes	61.1
	No	38.9

**Fig. 3:**
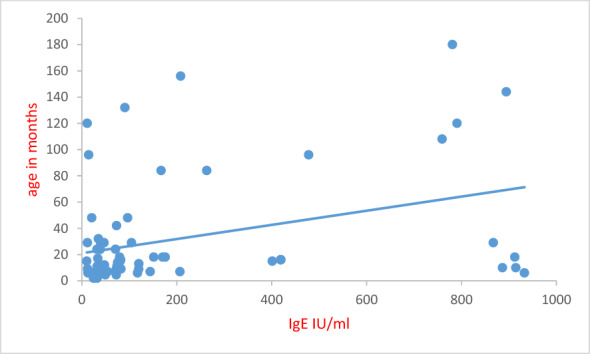
Correlation between serum IgE level and patient’s age

The mean IgE level of ARD patients were three times higher than control (187.9 vs 48.1 IU/ml); the difference was statistically significant (*p* = 0.015). Higher levels (201.2 and 203.2 IU/ml) were reported in patients who were early fed on both artificial milk and a solid food (*p* = 0.05). The level of IgE increases as the patient advances in age (*r* = 0.260, *p* = 0.03) (Fig. 3).

Early feeding, below 6 months of age, with a solid food was reported in 76% of ARD children, while artificial milk was reported in 79.8% of ARD differing from others (*p* < 0.001).

The risk assessment of early feeding in general with the development of ARD results in an odds ratio of 6.8 with 95% confidence interval (CI 3.1 to 15.3). Artificial milk early feeding associates with ARD development showed odds ratio of 6.1 with 95% confidence interval (CI 2.7 to 13.5). The solid food increases odds ratio by 1.6 with 95% CI 0.7 to 3.8. Table 4 lists the detailed assessment of association between these factors and the development and clinical features of ARD. Generally, early feeding is associated with a statistical significance with wheezing (OR 3.1, 95% CI 1.3 to 7.2), asthma (OR 4.8, 95% CI 2.2 to 10.6), itching (OR 3.1, 95% CI 1.2 to 8.1) and other allergy development (OR 5.8, 95% CI 2.0 to 16.4). Artificial milk increases wheezing (OR 4.3, 95% CI 1.9 to 9.4) and asthma (OR 7.6, 95% CI 3.5 to 16.3) with a statistically significant association. Allergenic nature of the artificial milk may be the principal cause. Solid food increases asthma (OR 4.1, 95% CI 1.8 to 9.7) and itching (OR 3.4, 95% CI 1.4 to 8.1) with a statistically significant association.

**Table 4:** Association of early feeding, solid food and artificial milk with the development of ARD and other clinical data among child patients attending child and maternity hospital in Ibb city, Yemen

Clinical data	Early feeding		Artificial milk		Solid food	
	Odds ratio	95% CI	Odds ratio	95% CI	Odds ratio	95% CI
ARD	6.8	3.0–15.3	6.1	2.7–13.5	1.6	0.7–3.8
Wheezing	3.0	1.3–7.2	4.3	1.9–9.4	1.9*	0.9–4.1
Asthma attack	4.8	2.2–10.5	7.6	3.5–16.3	4.2	1.8–9.7
Recurrent itchy	3.1	1.2–8.1	1.4*	0.6–3.2	3.4	1.5–8.1
Other allergies	5.8	2.1–16.5	3.1	1.3–7.4	1.8*	0.8–4.2
Admission to hospital	3.2	1.5–6.7	2.2	1.1–4.7	2.3*	1.0–4.9
Nebulizer use	2.6	1.2–5.6	2.1*	1.0–4.4	2.2*	1.0–4.9
Medication	4.9	2.2–10.8	4.8	2.2–10.4	2.6	1.2–5.6

^*^These results showed non-significant association (*p*  > 0.05)

## Discussion

Global public health recommendation states that infants should be exclusively breastfed for the first 6 months of life to achieve optimal growth, development and health [14]. However, most families in Yemen Republic believe that mother's milk is not sufficient to meet the nutritional requirements of their infants. They supply infant with other complementary formula such as artificial milk, juices and solid foods. Recommendations also state that infants should receive nutritionally adequate and safe complementary foods while breast-feeding continues for up to 2 years of age or beyond. Exclusive breast-feeding from birth is essential and possible except for a few medical conditions, and unrestricted exclusive breast-feeding results in ample milk production and consumption [16].

Our hypothesis about the predisposition of early infant feeding on the development of ARD mainly asthma was a matter of debate for many years ago. Most studies agreed with our results. More than that, some of them prove a protective effect of exclusive, and prolong breast-feeding from development of certain ARD and other atopic outcomes [17–19].

By contrast, a study in Belorussia concluded that there is no reduction in risk or even an increase in risk with breast-feeding [20]. However, Guilbert et al. concluded that a longer duration of breast-feeding favorably influences lung growth in children. But in the presence of maternal asthma, longer breast-feeding is associated with decreased airflows [21]. This indicates the superiority of genetic factor on the environmental one in the development of asthma. Breast-feeding affects lung health through some mechanisms, including epigenetic effects, modulation of gut microbiota, stimulation of lung growth and immune development [22].

Our results indicate to increased development of ARD in early fed infants mainly with artificial milk with OR of 6.8 and 6.1, respectively. In addition, early feeding is strongly associated with asthma attack (OR = 4.8), the artificial milk feeding is strongly associated with asthma attack (OR = 7.6) and wheezing (OR = 4.3). Camille et al. report association between wheezing and early feeding within French infants with OR of 3.78 [23]. Also Yu et al. report OR of 1.27 within Chinese children [24]. Moreover, it was found elevated risk of asthma at age 6 within children consuming meat at the first year of live by an OR of 8.47 [25]. Previously, a study concluded that an introduction of milk other than breast milk during the first 6 months after birth increased almost two fold the risk of development of persistent asthma [Adjusted Relative Risk (ARR): 1.71] [26].

In contrast, Greer et al. showed that there is no evidence that delaying the introduction of allergenic foods, including peanuts, eggs, and fish, beyond 4–6 months prevents atopic disease [27].

Other environmental factors may also affect the development of allergy. For example, living in rural areas increases exposure to the plant and dust allergens. Similarly, living at the ground floor also does [28]. Such factors were found to be higher within our patients. An exposure to allergen in utero is a suggested mechanism for early presentation of ARD symptoms because the presence of major respiratory allergens (Der p 1 and Blo t 5) in paired colostrum and cord blood samples [29]. Therefore, the symptoms appear early in the patient’s life. Half of our patients suffered from severe symptoms to the degree that need medical assistant, oxygen supplement and bronchodilator. Also, the most prevalent symptom was wheezing in accordance with another published data [30].

Allergic parents factor beside the dietary factors increase the risk of ARD development [31].

Most of the early fed patients exhibit higher level of IgE in their serum. The level of IgE increased with advance in age. This may denote the induction potential of early feeding on ARD development and consequently on symptoms presentation as exhibited by the direct correlation between age of artificial milk feeding and the age of symptoms presentation. This induction is reflected also by the elevation of eosinophil, lymphocytes and platelets among patients rather than healthy control.

## Conclusions

Our result confirms that the early infant feeding, particularly artificial milk predispose infant to the development of allergic respiratory disease with more frequent clinical features of wheezing and asthma attack. Other sensitization markers such as elevated eosinophil count and serum IgE level noted in the early fed children confirm the development of allergic disease. Exclusive breast-feeding for the first 6 months of age seemed to be the important risk reduction behavior to manage ARD burden in infants and children.

## Data Availability

All data are available in this manuscript.

## Abbreviations

ARD: Allergic respiratory diseases
IgE: Immunoglobulin E
OR: Odds ratio
CI: Confidence interval
EDTA: Ethylene diamine tetra acetic acid
ELISA: Enzyme linked immunosorbent assay

## References

[1] Asher MI, Montefort S, Bjorksten B, Lai CK, Strachan DP, Weiland SK, Williams H (2006). ISAAC phase three study group. Worldwide time trends in the prevalence of symptoms of asthma, allergic rhinoconjunctivitis, and eczema in childhood: ISAAC phases one and three repeat multicountry cross-sectional surveys. Lancet.

[2] Bahaj S, Moharem A, Kaid A (2012). Prevalence of asthma and allergic diseases among high school students in urban and rural communities, Yemen. Egypt J Med Microbiol.

[3] Masjedi M, Ainy E, Zayeri F, Paydar R (2018). Assessing the prevalence and incidence of asthma and chronic obstructive pulmonary disease in the Eastern Mediterranean Region. Turk Thorac J.

[4] Nwaru BI, Hickstein L, Panesar SS, Muraro A, Werfel T, Cardona V, Dubois AE, Halken S, Hoffmann-Sommergruber K, Poulsen LK (2014). The epidemiology of food allergy in Europe: a systematic review and meta-analysis. Allergy.

[5] Dierick BJH, van der Molen T, Flokstra-de Blok BMJ, Muraro A, Postma MJ, Kocks JWH, van Boven JFM (2020). Burden and socioeconomics of asthma, allergic rhinitis, atopic dermatitis and food allergy. Expert Rev Pharmacoecon Outcomes Res.

[6] Flokstra-de Blok BMJ, Dubois AEJ, Vlieg-Boerstra BJ, Oude Elberink JNG, Raat H, DunnGalvin A, Hourihane JB, Duiverman EJ (2010). Health-related quality of life of food allergic patients: comparison with the general population and other diseases. Allergy.

[7] Mallol J, Crane J, Mutius E, von J Odhiambo U Keil A Stewart,  (2012). ISAAC phase three study group. The International Study of Asthma and Allergies in Childhood (ISAAC) phase three: a global synthesis. Allergol Immunopathol (Madr).

[8] Gupta R, Sheikh A, Strachan DP, Anderson HR (2007). Time trends in allergic disorders in the UK. Thorax.

[9] Chesnutt AN, Chesnutt MS, Prendergast NT, Prendergast TJ, Papadakis MA, McPhee SJ, Rabow MW (2021). Pulmonary disorders. Current medical diagnosis & treatment.

[10] van Neerven RJJ, Savelkoul H (2017). Nutrition and allergic diseases. Nutrients.

[11] Greer FR, Sicherer SH, Burks AW (2008). Effects of early nutritional interventions on the development of atopic disease in infants and children: The role of maternal dietary restriction, breastfeeding, timing of introduction of complementary foods, and hydrolyzed formulas. Pediatrics.

[12] Klopp A, Vehling L, Becker AB, Subbarao P, Mandhane PJ, Turvey SE, Lefebvre DL, Sears MR, Azad MB (2017). Modes of infant feeding and the risk of childhood asthma: a prospective birth cohort study. J Pediatr.

[13] Ip S, Chung M, Raman G, Chew P, Magula N, DeVine D, Trikalinos T, Lau J (2007). Breastfeeding and maternal and infant health outcomes in developed countries. Evid Rep Technol Assess (Full Rep).

[14] World Health Organization (2008) Indicators for assessing infant and young child feeding practices: part 1: definitions: conclusions of a consensus meeting held 6–8 November 2007 in Washington D.C., USA. World Health Organization: Geneva.https://apps.who.int/iris/handle/10665/43895

[15] Jones KD, Berkley JA, Warner JO (2010). Perinatal nutrition and immunity to infection. Pediatr Allergy Immunol.

[16] World Health Organization & United Nations Children's Fund (‏UNICEF) (2003) Global Strategy for Infant and Young Child Feeding. World Health Organization: Geneva.https://apps.who.int/iris/handle/10665/42590

[17] Saarinen UM, Kajosaari M (1995). Breastfeeding as prophylaxis against atopic disease: prospective follow-up study until 17 years old. Lancet.

[18] Dell S, To T (2001). Breastfeeding and asthma in young children. Arch Pediatr Adolesc Med.

[19] Kull I, Almqvist C, Lilja G, Pershagen G, Wickman M (2004). Breast-feeding reduces the risk of asthma during the first 4 years of life. J Allergy Clin Immunol.

[20] Kramer MS, Vanilovich I, Platt R, Bogdanovich N, Sevkovskaya Z, Dzikovich I, Shishko G, Mazer B (2007). Effect of prolonged and exclusive breast feeding on risk of allergy and asthma: cluster randomised trial. BMJ.

[21] Guilbert TW, Stern DA, Morgan WJ, Martinez FD, Wright AL (2007). Effect of breastfeeding on lung function in childhood and modulation by maternal asthma and atopy. Am J Respir Crit Care Med.

[22] Miliku K, Azad MB (2018). Breastfeeding and the developmental origins of asthma: current evidence, possible mechanisms, and future research priorities. Nutrients.

[23] Davisse-Paturet C, Raherison C, Adel-Patient K, Divaret-Chauveau A, Bois C, Dufourg MN, Lioret S, Charles MA, de Lauzon-Guillain B (2019). Use of partially hydrolysed formula in infancy and incidence of eczema, respiratory symptoms or food allergies in toddlers from the ELFE cohort. Pediatr Allergy Immunol.

[24] Yu B, Dai L, Chen J, Sun W, Chen J, Du L, Deng N, Chen D (2019). Prenatal and neonatal factors involved in the development of childhood allergic diseases in Guangzhou primary and middle school students. BMC Pediatr.

[25] Hose AJ, Pagani G, Karvonen AM, Kirjavainen PV, Roduit C, Genuneit J, Schmaußer-Hechfellner E, Depner M, Frei R, Lauener R, Riedler J, Schaub B, Fuchs O, von Mutius E, Divaret-Chauveau A, Pekkanen J, Ege MJ (2021). Excessive unbalanced meat consumption in the first year of life increases asthma risk in the PASTURE and LUKAS2 birth cohorts. Front Immunol.

[26] El-Heneidy A, Abdel-Rahman ME, Mihala G, Ross LJ, Comans TA (2018). Milk other than breast milk and the development of asthma in children 3 years of age a birth cohort study (2006–2011). Nutrients.

[27] Greer FR, Sicherer SH, Burks W (2019). The effects of early nutritional interventions on the development of atopic disease in infants and children: the role of maternal dietary restriction breastfeeding hydrolyzed formulas, and timing of introduction of allergenic complementary foods. Pediatrics.

[28] Deng SZ, Jalaludin BB, Antó JM, Hess JJ, Huang CR (2020). Climate change, air pollution, and allergic respiratory diseases: a call to action for health professionals. Chin Med J.

[29] Macchiaverni P, Ynoue LH, Arslanian C, Verhasselt V, Condino-Neto A (2015). Early exposure to respiratory allergens by placental transfer and breastfeeding. PLoS ONE.

[30] Kull I, Wickman M, Lilja G, Nordvall SL, Pershagen G (2002). Breast feeding and allergic diseases in infants—a prospective birth cohort study. Arch Dis Child.

[31] Dick S, Friend A, Dynes K, AlKandari F, Doust E, Cowie H, Ayres JG, Turner SW (2014). A systematic review of associations between environmental exposures and development of asthma in children aged up to 9 years. BMJ Open.

